# The Rôle of Flies in the Spread of Dysentery

**Published:** 1918-05

**Authors:** 


***The Rôle of Flies in the Spread of Dysentery.*** By E. ROUBAUD, *Bulletin de la Société de Pathologie Exotique*, March, 1918, N° 3, p. 166.

The author agrees with Kuenen and Swellengrebel that flies are not likely to act as agents in disseminating protozoic cysts of intestinal parasites, especially of amoebae. He finds that direct transmission by the soiling of the fly's body is practically out of the question. He finds also that flies are incapable of propagating free amoebae in their intestines. On the contrary, flies may excrete cysts of intestinal protozoa, but unless these are deposited on liquids or moist matter the drying of the cyst in the faeces of the fly is so rapid as to cause almost immediate death of the organism. The writer is inclined to think that in this manner the fly tends rather to diminish than to increase the spread of the infection.

